# Activity and molecular targets of pioglitazone via blockade of proliferation, invasiveness and bioenergetics in human NSCLC

**DOI:** 10.1186/s13046-019-1176-1

**Published:** 2019-04-26

**Authors:** Vincenza Ciaramella, Ferdinando Carlo Sasso, Raimondo Di Liello, Carminia Maria Della Corte, Giusi Barra, Giuseppe Viscardi, Giovanna Esposito, Francesca Sparano, Teresa Troiani, Erika Martinelli, Michele Orditura, Ferdinando De Vita, Fortunato Ciardiello, Floriana Morgillo

**Affiliations:** 1 Medical Oncology, Department of Precision Medicine, University of Campania “Luigi Vanvitelli”, Via Pansini 5, 80138 Naples, Italy; 2 Department of Medicine, Surgery, Neurology, Metabolism and Geriatrics School of Medicine and Surgery, University of Campania “Luigi Vanvitelli”, Naples, Italy

**Keywords:** Glitazones, Metabolism, Lung cancer, EMT, Bioenergetics

## Abstract

**Background:**

Pioglitazone, a synthetic peroxisome proliferator activated receptor (PPAR-γ) ligand, is known as an antidiabetic drug included in the thiazolidinediones (TZDs) class. It regulates the lipid and glucose cell metabolism and recently a role in the inhibition of numerous cancer cell processes has been described.

**Methods:**

In our work we investigate the anti-tumor effects of pioglitazone in in vitro models of non small cell lung cancer (NSCLC) and also, we generated ex-vivo three-dimensional (3D) cultures from human lung adenocarcinoma (ADK) as a model to test drug efficacy observed in vitro. The inhibitory effect of pioglitazone on cell proliferation, apoptosis and cell invasion in a panel of human NSCLC cell lines was evaluated by multiple assays.

**Results:**

Pioglitazone reduced proliferative and invasive abilities with an IC_50_ ranging between 5 and 10 μM and induced apoptosis of NSCLC cells. mRNA microarray expression profiling showed a down regulation of MAPK, Myc and Ras genes after treatment with pioglitazone; altered gene expression was confirmed by protein analysis in a dose-related reduction of survivin and phosphorylated proteins levels of MAPK pathway. Interestingly mRNA microarray analysis showed also that pioglitazone affects TGFβ pathway, which is important in the epithelial-to-mesenchimal transition (EMT) process, by down-regulating TGFβR1 and SMAD3 mRNA expression. In addition, extracellular acidification rate (ECAR) and a proportional reduction of markers of altered glucose metabolism in treated cells demonstrated also cell bioenergetics modulation by pioglitazone.

**Conclusions:**

Data indicate that PPAR-γ agonists represent an attractive treatment tool and by suppression of cell growth (in vitro and ex vivo models) and of invasion via blockade of MAPK cascade and TGFβ/SMADs signaling, respectively, and its role in cancer bioenergetics and metabolism indicate that PPAR-γ agonists represent an attractive treatment tool for NSCLC.

## Background

Glitazones, also known as thiazolidinediones (TZDs), are a group of antidiabetic drugs commonly used as therapeutic agents for the treatment of type 2 diabetes mellitus (T2MD). Chemically, they derivate from the parent compound thiazolidinedione, and include pioglitazone and rosiglitazone still used as mono-therapy or in combination with other oral agents, such as metformin or sulphonylureas and other experimental, failed or non-marketed agents such as troglitazone (withdrawn from the market due to idiosyncratic liver adverse events), ciglitazone, darglitazone or englitazone.

TZDs have potent insulin-sensitizing activity used to improve lipid and glucose metabolism through the modulation of peroxisome proliferator-activated receptors, PPARs [1]. These are members of a nuclear receptor superfamily that regulate nutrient-dependent transcription first identified in the 1990s in rodents and named after their property of peroxisome proliferation [2]. Similar to other nuclear receptor family members, all of them have a canonical domain structure formed by an amino-terminal region with a DNA binding domain, a ligand-independent trans-activation domain (AF1) and a carboxyl-terminal region that consists in a dimerization and ligand-binding domain with a ligand-dependent trans-activation region (AF2) [3, 4].

Recently, the role of PPARs have been reconsidered as they regulate not only cell bioenergetics but are also involved in cell proliferation, apoptosis and tumorigenesis [5]. Functional activity of PPARs in cancer is related to their modulation of phosphates and kinases, including ERK1/2, P38-MAPK, PKC, AMPK, and GSK3 and although all PPAR isoforms are implicated in several metabolic syndromes, PPAR-γ seems to be mostly involved in tumorigenesis regulation via activation of these pathways [6]. Previous papers have shown that PPAR-γ is expressed in different tumor cells such colon, breast and lung cancer cell lines and the activation of PPAR-γ by ligands led to either inhibition of cell growth or induction of apoptosis [7, 8]. For example, Lv et al demonstrated that PPARγ activation induced cell cycle G2 arrest and inhibition of bladder cancer cells proliferation in vitro involving the inhibition of PI3K-Akt pathway [9].

PPAR-γ activation have been found to inhibit the growth lung cancer cell lines through an increase in apoptosis. For example, ciglitazone inhibited growth of lung cancer cell H1650 in the time- and dose-dependent manner stimulating caspase 3/7 activity [10].

Previous studies concerning the inhibitory effect of PPAR-γ ligands on the metastatic potential of cancer cells have been reported [11, 12]. Despite these evidences, the role of TDZs in tumor biology remains unclear and few reported studies have investigated molecular pathways involved in the potential role of these PPAR-γ agonists as anti-cancer agents. The present work aimed to investigate whether pioglitazone, a prescription TZD class drug and a ligand of PPAR-γ, inhibits the proliferation and metastatization of human NSCLC cell lines.

## Material and methods

### Cell lines, drug and chemicals

The human NSCLC H1299, H460, A549, H1975, HCC827 and human bronchial epithelial Beas2B cell lines were provided by American Type Culture Collection (ATCC, Manassas, VA, USA) and maintained in RPMI 1640 (Sigma-Aldrich) medium supplemented with 10% fetal bovine serum (FBS; Life Technologies, Gaithersburg, MD) in a humidified atmosphere with 5% CO_2_. The identity of all cell lines was confirmed by STR profiling (Promega) on an ad hoc basis prior to performing experiments and repeated after the majority of the experiments were performed.

Pioglitazone was purchased from Sigma-Aldrich.For*in vitro* studies, pioglitazone was dissolved in sterile dimethylsulfoxide (DMSO) and the stock solution (10 mM) was stored in aliquots at − 20 °C. Working concentrations were diluted in culture medium just before each experiment.

Primary antibodies for western blot analysis against were obtained from Cell Signaling Technology; the following secondary antibodies from Bio-Rad were used: goat anti-rabbit IgG, rabbit anti-mouse IgG and monoclonal anti-α-tubulin antibody (T8203) from Sigma Chemical Co.

### Cell viability assay

Cells were seeded in 24-well plates at the density of 1 × 104 cells/well and were treated with increasing doses of pioglitazone from 0.1 μM to 50 μM for 72 h. Cell proliferation was measured with 3-(4,5-dimethylthiazol-2-yl)-2,5-diphenyltetrazoliumbromide (MTT, Sigma-Aldrich). The concentrations inhibiting 50% of cell growth (IC_50_) were obtained and the corresponding values were used for subsequent experiments. Results represent the median of three separate experiments, each performed in duplicate.

### Generation of ex vivo cultures from lung adenocarcinoma patient samples

We developed a protocol for ex vivo 3D cultures from patient adenocarcinoma (ADK) samples. The protocol has been approved by the local Ethics Committee of the University of Campania and all patients gave their written informed consent to the use of the tumor sample. All fresh tumor tissue samples were kept on ice and processed in sterile conditions on the day of collection. Tissue fragments were digested as previously described [13] in a 37 °C shaker at low to moderate speed (e.g. 200 rpm) for incubation time between 12 and 18 h and cells were separated with serial centrifugation. For 3D cultures, cells were seeded in Matrigel in order to preserve three dimensional structure.

### Colony forming assays

Colony forming assay was performed to evaluate the long-term proliferative potential H1299, H460 and Beas2B cells following treatment. Cells were seeded on 6-well tissue culture dishes at 300 cells/well and treated with indicated drug at different doses for 72 h. Cells were maintained for 14 days with fresh culture media every 3 days, at which point they were fixed with 4% paraphormaldeid at room temperature (RT) for 15 min, stained with 0.1% crystal violet and colonies counted using the ImageJ plugin. All conditions were performed in triplicate and untreated cells were used as control.

### Assessment of apoptosis

Apoptosis was evaluated by flow cytometry using AnnexinV-FITC and 7-Amino-Actinomicin D (7-AAD) double staining (Thermo fisher) according to the manufacturer’s instruction. The detection of viable cells, early and late apoptosis cells, and necrotic cells were performed by BD Accuri™ C6 (BD Biosciences) flow cytometer and subsequently analyzed by ACCURI C6 software (Becton Dickinson). Results represent the median of three separate experiments, each performed in duplicate.

### Quantitative real time PCR (qPCR)

Total RNA from cells was extracted using Trizol reagent (LifeTechnologies) according to the manufacturer’s instructions. The primers used to evaluate the expression levels of genes encoding for TGFβR1, SMAD3 and SMAD4 were: 5′-gcagcagacaataaagacaatgg-3’and 5′-tgctcatgataatctgacaccaacc-3′ for TGFβR1; 5′-cccatcccggacattactgg-3′ and 5′-atccaggagcaggatgattgg-3′ for SMAD4; 5′-gaacgtcaacaccaagtgcat-3’and 5′-acgcagacctcgtccttct-3′ for SMAD3; 5′-ggcgacgacccattcgaac-3′ and 5′-aggcacggcgactacctc-3′ for 18S.

All samples were run in duplicate, using the Mastercycler CFX-96 (Bio-Rad) and relative expression of genes was determined by normalizing to 18S, used as internal control gene. All assays included a melting curve analysis for which all samples displayed single peaks for each primer pair. To calculate the fold change in value it was used the 2- ΔΔCt method. Nonspecific signals caused by primer dimers were excluded by dissociation curve analysis and use of non-template controls. Data were then reported as mean fold change ± SD over the minimal value arbitrarily assigned to a reference sample and ANOVA followed by Duncan’s test for multigroup comparison was carried out to assess the significance of differences.

### Western blot analysis

H1299 and H460 cancer cells were seeded into 100 mm^3^ petri dishes and treated for 24 h with pioglitazone [1 and 10 μM]. Protein lysates were obtained by homogenization in RIPA lysis buffer (Sigma-Aldrich, MO, USA) with protease and phosphatase inhibitors cocktail (Hoffmann-La Roche). Protein extracts were quantified by using Bradford assay (Bio-Rad, CA, USA) and equal amounts of total protein (40 μg/lane) were separate by 4–15% gradient mini precast TGX gel (Bio-Rad) before transferring to nitrocellulose by standard western conditions, blocked in BSA solution and primary antibodies (1:1000 in BSA solution; incubated overnight at 4 °C). The secondary antibody (1:3000 in 5% milk/TBS/Tween20 solution) was incubated at RT for 1 h before detection. Immunocomplexes were detected with the enhanced chemiluminescence kit ECL plus, by Thermo Fisher Scientific (Rockford,IL) using the ChemiDoc (Bio-Rad). Values were normalized to α-tubulin. Each experiment was done in duplicate.

### Microarray gene expression analysis

The NSCLC cells, H1299 and H460, were seeded in 100 mm^3^ petri dishes until reaching a confluence of 70% and treated with pioglitazone [10 μM] for 24 h. Then they were collected by scraping for RNA extraction and subsequent microarray analysis. Total RNA (200 ng) isolated from the cells was amplified and labeled with cyanine 3 (Cy3) for use in generating complementary RNA (cRNA) with Low Input Quick Amp LabelingKit, one-color (Agilent, Santa Clara, CA, USA). The labeled cRNA samples were hybridized to the Human Gene Expression Microarray 8 × 60 K (Agilent) consisting of 26,083 unique Entrez Genes, 30,606 unique lncRNA, and positive and negative controls for the monitoring of auto and cross-hybridization. For each array, 600 ng of Cy3-labeled, linearly amplified cRNA were used for hybridization using Gene Expression Hybridization Kit (Agilent). Hybridized slides were scanned using MS200 Roche scanner. The array was scanned to ensure that less than 1% of the total number of spots had median intensities near the maximum. Images were analyzed using Feature Extraction 12.5 image analysis software (Agilent). After transformation of the data into log, the normalization was carried out by 75th percentile, the filtering of the probes on the basis of the expression values and the flags to retain only the probes that exceed the quality criteria using GeneSpring GX 14.5 software (Agilent). A Principal Component Analysis (PCA) was performed on the dataset of values and on samples. Only a fold change analysis was performed applying a cut-off> 1.5. The analysis was performed vs zero, not having a paired check. A statistical analysis could not be performed because there were no replicates of the samples. The analysis of the pathways of the differentially expressed genes was performed on the following databases: Wiki, BioCyc and Kegg.

### SeaHorse analysis

Glycolysis was measured with a XF-96 extracellular flux analyzer SeaHorse. According to manufacturer’s recommended protocol of Seahorse XF96 extracellular flux analyzer, cell medium was replaced by the conditional medium and incubated at the incubator without supplied CO_2_ for one hour before completion of probe cartridge calibration. Extracellular acidification rate (ECAR) was measured in the Seahorse XF96 Flux analyzer. Measurements were performed after injection of three compounds affecting bioenergetics: glucose, oligomycin and 2DG (Sigma, St Louis, MO, USA) and the measurements were normalized against the cell densities. Each experiment contained triplicate data points.

### Statistical analysis

Statistical analyses of the in vitro data were performed using a one-way analysis of variance (ANOVA). Quantitative data were reported as mean ± Standard Deviation (SD) from three or more independent experiments. Results were compared by analysis of variance (ANOVA) followed by the Student t-test.

## Results

### Effect of pioglitazone on proliferation, migration and apoptosis in human NSCLC cell lines

**Fig. 1:**
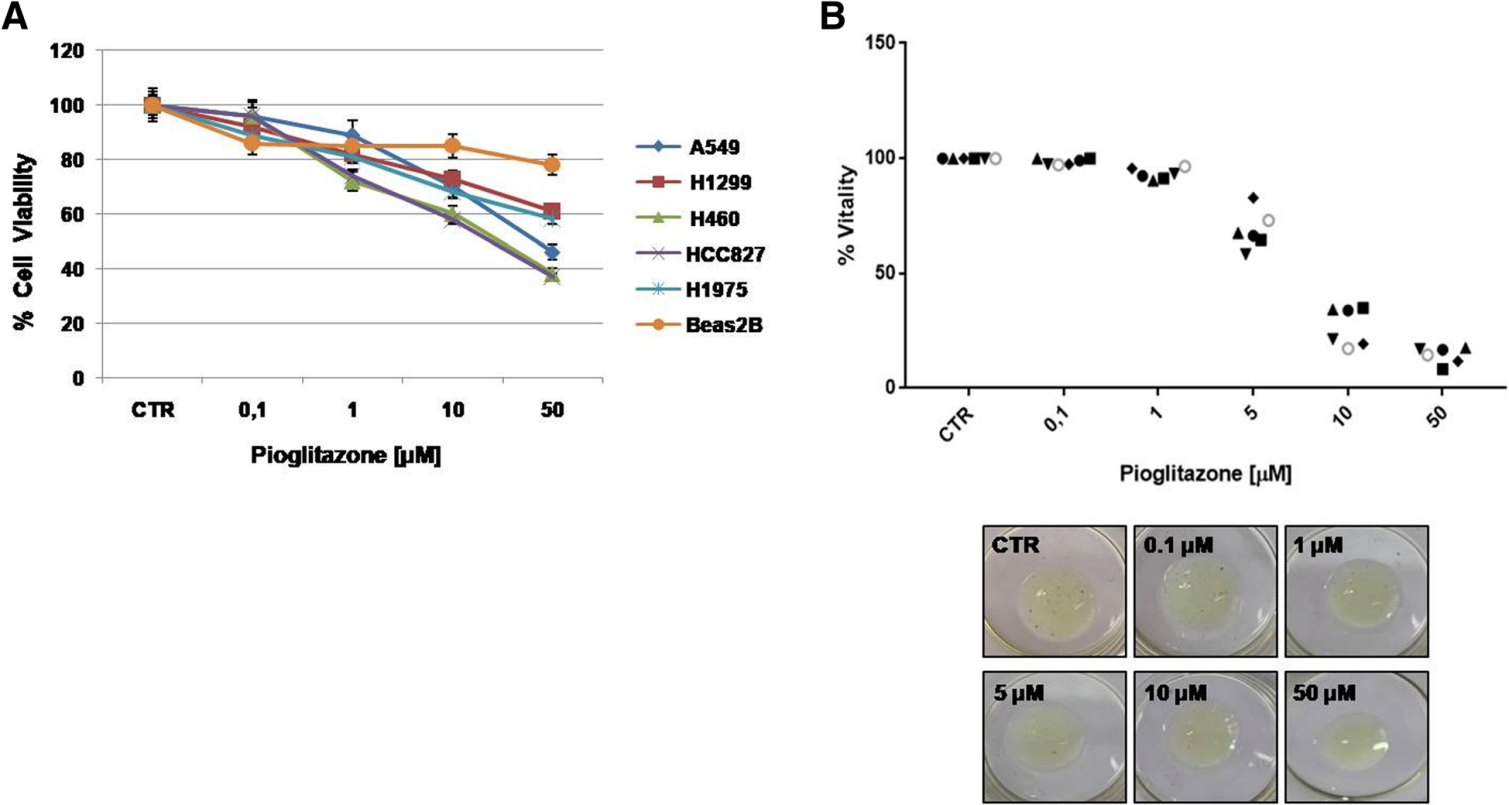
Effect of pioglitazone treatment on cell proliferation in a panel of human lung cancer and non-tumor cell lines, and ADK patient samples. Cells were treated with different concentrations of pioglitazone (range 0.1–50 μM) for 72 h in in vitro (**a**) and 3D culture from ADK patient samples, symbols indicate different patient samples (**b**). The images shown are representative of one of the experiments conducted on organoids deriving from ADK patient samples seeded in Matrigel and photographed after treatment with pioglitazone. The proliferation rate was evaluated by MTT assay, as described in Materials and Methods. Data are the average ± SD of three independent experiments, each performed in triplicate. Asterisks indicate statistical significance, *P* < 0.01

Pioglitazone, along its ability of suppress oxidative stress and damage through improving antioxidant capacity, has been reported to have a role in suppression of MAPK, Nf-kB signaling pathways and inhibition of TNFα with an IC_50_ dose of 1.61 M at 24th hour and 2.85 M at 48th hour [14, 15]. In order to evaluate the anti-proliferative effect of pioglitazone as single agents, we used several human non small lung cancer (NSCLC) cell lines: A549, H1299, H460, H1975, HCC827 and human bronchial epithelial cells, Beas2B, as control. Cell proliferation was measured with the 3-(4,5- dimethylthiazol-2-yl)-2,5 diphenyltetrazolium bromide (MTT) assay in a dose-response experiment in which cells were cultured in presence of increasing doses of pioglitazone from 0.1 μM to 50 μM. After 72 h treatment we observed a progressive decrease in cell proliferation with the higher doses of 50 μM allowing only 20–30% of cells surviving. All cell lines behaved similarly showing an IC_50_ of about 10 μM with the exception of Beas2B that were not sensitive to treatment demonstrating the efficacy of pioglitazone only on cancer cells (Fig. 1a).

**Fig. 2:**
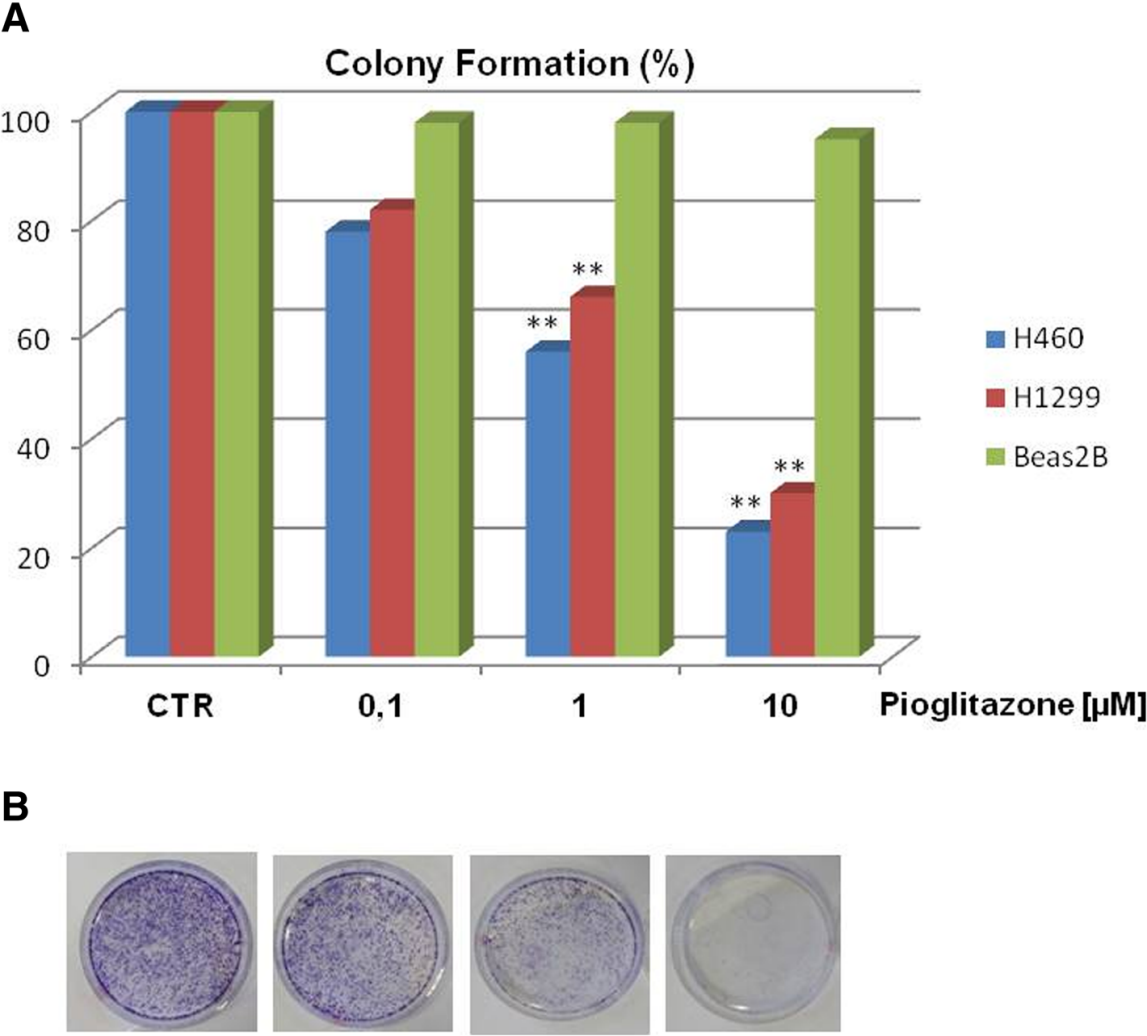
Effect of pioglitazone treatment on colony formation in H1299 and H460 lung cancer cells and Beas2B normal cell line. Cells were treated with pioglitazone (range 0.1–10 μM) for 72 h. Colonies were stained with 0.1% crystal violet and counted as described in Materials and Methods. a Histogram of colony number counted by imageJ plugin. Asterisks indicate statistical significance, *P* < 0.05. **b** Representative images of lung cancer cells (H460) acquired by phase-contrast microscope

We have established in parallel ex vivo 3D culture from surgical samples of ADK patients to estimate the feasibility of both approaches (in vitro and ex vivo). The ex vivo growth capacity of cultures derived from the patient came from six samples analyzed one week after seeding in Matrigel. Therefore, we tested the effect of pioglitazone at the same dose used in vitro and prolonged the treatment for one week. The results shown in the Fig.1b demonstrated that the inhibition of proliferation by pioglitazone is also significant in several models. However, we have deepened these mechanisms by focusing on the NSCLC that represent a more suitable model for the different experimental approaches. To confirm the anti-proliferative ability of the drug and to evaluate the abilities of cells to form colonies in vitro in the presence and absence of pioglitazone, we performed colony forming assays. Similarly, pioglitazone significantly affected the colony-forming abilities of all NSCLC cell lines in a dose-dependent manner with a peak of 80% of reduction at the dose of 10 μM (Fig. 2a). On the contrary, in the control group, cancer cells formed large cell cluster within 24–48 h confirming that cells maintained their invasive and migratory abilities in the absence of treatment. Anyway, these data indicate that the effect of pioglitazone is mainly linked to cell colonies formation and consequently to the capacity of cells to reach the surrounding microenvironments and form new tumor sites. The same treatment performed on Beas2B cells showed no effect (Fig. 2a): the presence of pioglitazone didn’t alter the ability to form colonies in normal cells suggesting that the drug has a selective action on tumor cells and for this reason we have chosen to take advantage of this aspect by focusing on cancer cells.

**Fig. 3:**
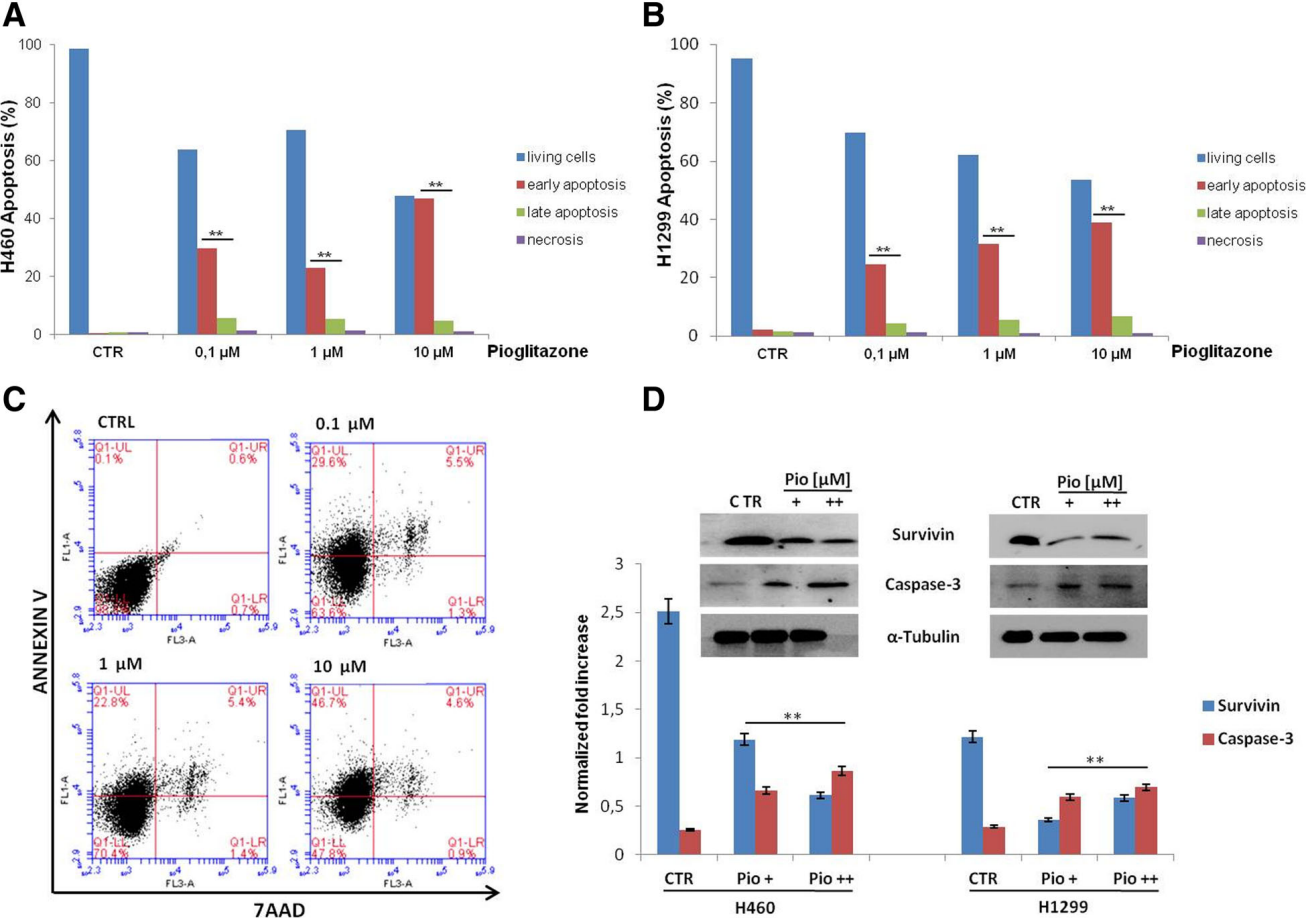
Effect of pioglitazone treatment on induction of apoptosis in H1299 and H460 lung cancer cells. **a**, **b** Histogram of data expressed as percentage of apoptotic cells. Bars represents mean values obtained from three separate experiments. *P* values < 0.05 were considered as statistically significant (**). **c** Representative flow cytometric analysis of H460 cell apoptosis. One representative experiment is shown. Dot plots diagrams show the different stages of apoptosis. % indicated in the upper left quadrant represent cells positive for annexin V and negative for 7AAD, considered as apoptotic cells; % in upper right quadrant indicate cells positive for both annexin V and 7AAD, showing the late apoptotic or necrotic cells population; % in lower left quadrant are negative for both markers and represent viable cells. **d** Expression of caspase-3 and survivin evaluated by immunoblotting as described in Materials and Methods with indicated pioglitazone treatments: +: 1 μM; ++: 10 μM. α-tubulin was used as the loading control

Moreover, as an increase in anti-proliferative effects are usually associated with an increase in apoptotic rate, we measured the ability of pioglitazone to induce apoptosis by using Annexin V-FITC assay. As depicted in Fig. 3a, compared to the control untreated group, the treatment with pioglitazone at the dose of 0.1, 1 and 10 μM induced a significant increase in early and late apoptosis within 48 h of treatment in H1299 and H460 of human NSCLC. In particular, H460 and H1299 cells showed an apoptotic rate in the range of 30–35% with 0.1–1 μM treatment dose; while at the dose of 10 μM cells reached an apoptotic rate of 51 and 46% in H460 and H1299 respectively (*P* < 0.05, Fig. 3a, b).

To further investigate the anti-proliferative and pro-apoptotic effects of pioglitazone, the activation status of different pro- and/or anti-apoptotic factors was examined by western blot analysis done on protein extracts from H460 and H1299 cells that were treated with 1 and 10 μM of pioglitazone (Fig. 3c). We observed increasing expression levels of cleaved caspase-3 suggesting an activation of extrinsic apoptotic mechanism and a reduction of survivin indicating that pioglitazone seems to exert an anti-cancer effect by modulating both survival and apoptotic markers.

### Analysis of gene and protein expression profiles in NSCLC treated with pioglitazone

**Fig. 4:**
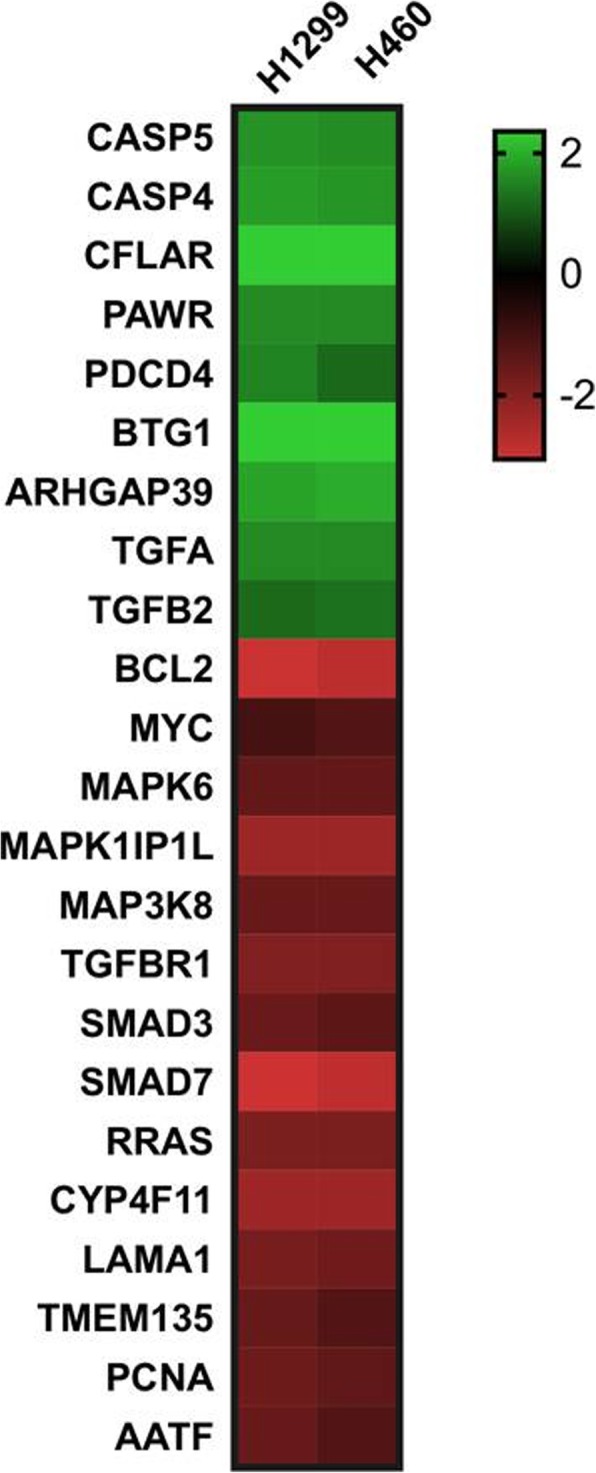
Effect of treatment with pioglitazone on mRNA expression. Heatmap represents list of up regulated and down regulated genes in NCSLC cell lines, in H1299 and H460, pioglitazone treatment vs untreated control

Activation of the classic cellular mechanisms, including MAPK cascade and other key regulators of cell life/death, represents a common event in cancer and some genes within this pathway are usually mutated or aberrantly expressed. In order to identify target genes whose expression may be significantly affected by pioglitazone treatment, we conducted microarray analysis on genes involved in cancer related pathways before and after treatment with pioglitazone. In particular, we performed an in vitro treatment with pioglitazone on lung cancer cell lines, and the extracted mRNAs were used for gene expression analysis. Among the total of screened genes, we identified 23 genes of interest as up-regulated or down-regulated, respectively, in pioglitazone treated cells compared to untreated control cells (cut-off for fold change > 1.5). As reported in Fig. 4, among the differentially expressed genes and consistently with our previous data, we observed the up regulation of genes involved in apoptosis (i.e. CASP5, CASP4, CFLAR, PAWR) and anti-proliferative signals (i.e. PDCD4, BTG1). On the contrary, among the down regulated genes, we found canonical genes involved in cell proliferation cascades, such as Myc, R-Ras, MAPK6, MAP3K8, as well as Bcl-2, PCNA and laminin. Of interest, gene expression of the SMADs family and the related TGFβ receptors, that are generally involved in the mechanism that regulates the epithelial-to-mesenchymal transition (EMT), resulted significantly decreased, suggesting that in addition to inhibition of proliferation and induction of apoptosis, the antimetastatic effects exerted by pioglitazone, as indicated by colony-forming and migration assays, could be explained by the ability of the drug to affect TGFβ pathway gene expression. TGFβ is a family of dimeric polypeptide growth factors that initiate cell signaling by dimerizing the TGFβ type 1 and type 2 serine/threonine kinase receptors, TGFβR1 and TGFβR2. This dimerization and phosphorylation propagates the signal by activating the intracellular SMADs transducers. Alteration of some of the components of this pathway has been intimately linked to the control of cell proliferation and differentiation in many cancer diseases; indeed TGFβ plays a key role in the tumor progression and metastatization of many types of tumor cells, which suggests that TGFβ signaling has tumor promoting effects in advanced disease.

**Fig. 5:**
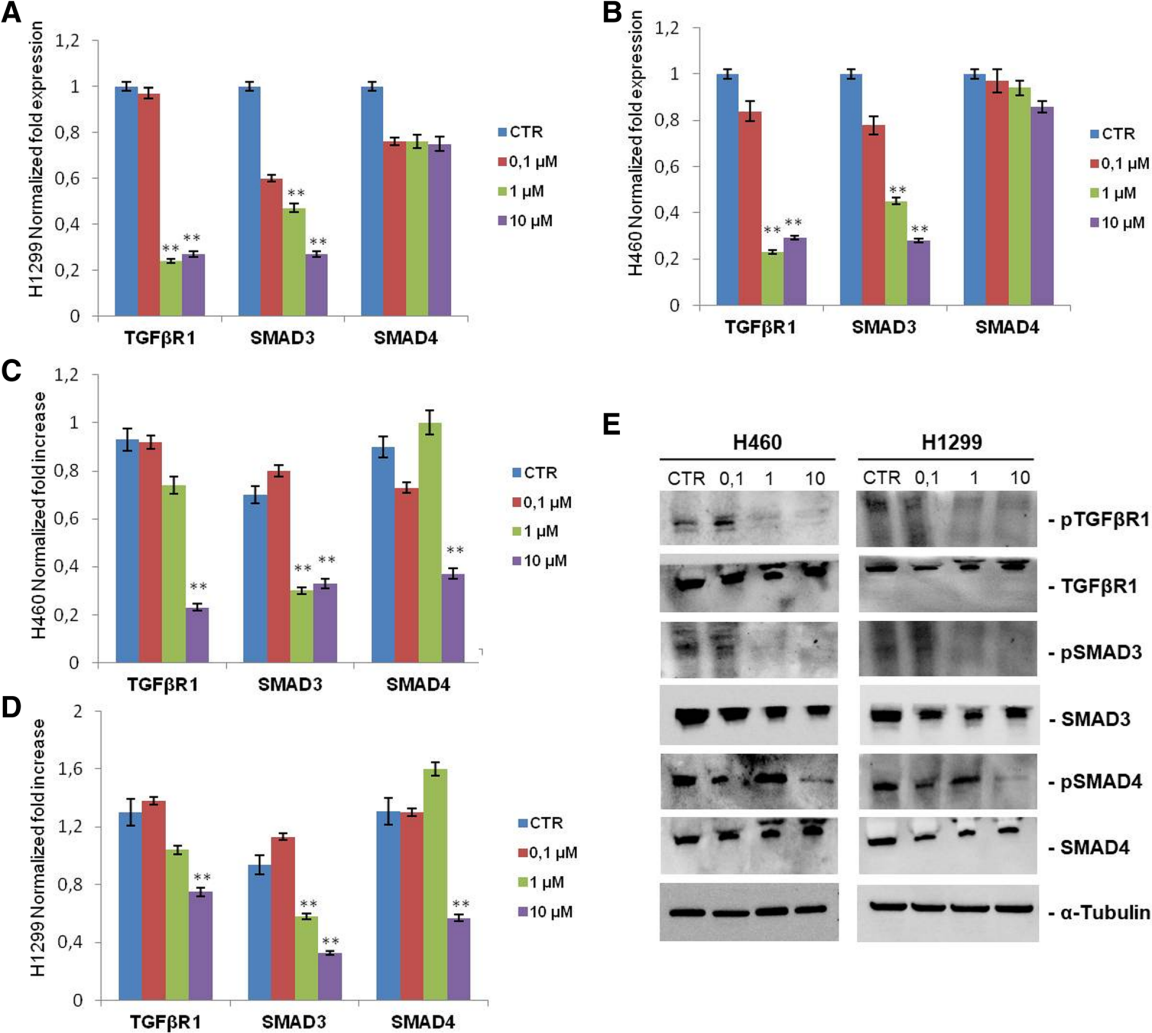
Effect of pioglitazone on TGFβ/SMADs pathways in H1299 and H460 lung cancer cells. **a**, **b** qPCR expression analysis of total mRNA from lung cancer cells, H1299 (**a**) and H460 (**b**). Gene expression levels was determined by normalizing to 18S. *P* values < 0.05 were considered as statistically significant (**). **c-e** Western blot analysis of TGFβ/SMADs protein expression levels in lung cancer cells, H460 and H1299

To better investigate the effects of pioglitazone on TGFβ pathway, we analyzed mRNA and protein levels of TGFβR1 and SMAD messengers in H460 and H1299 human NSCLC cell lines treated with different doses of drug (Fig. 5). The analysis of mRNA expression by qPCR revealed a significant reduction in the expression of TGFβR1 and SMAD3 in both cell lines. This effect is statistically significant and correlates with increasing doses of pioglitazone; in particular, at 1 and 10 μM a 4 fold-reduction of TGFβR1 and SMAD3 mRNA expression levels, respectively, was observed compared to untreated control. The same treatment does not seem to have effect on the expression level of SMAD4 which is recognized as an independent cofactor not directly in the TGFβR1/SMAD3 signaling cascade (Fig. 5a, b). Even at protein levels, by western blot analysis, we observed a strong decrease in TGFβR1 and SMADs expression levels with maximum effect at the doses 1 and 10 μM, the latter seems to be able to turn off all the TGFβ signaling cascade (Fig. 5c-e).

**Fig. 6:**
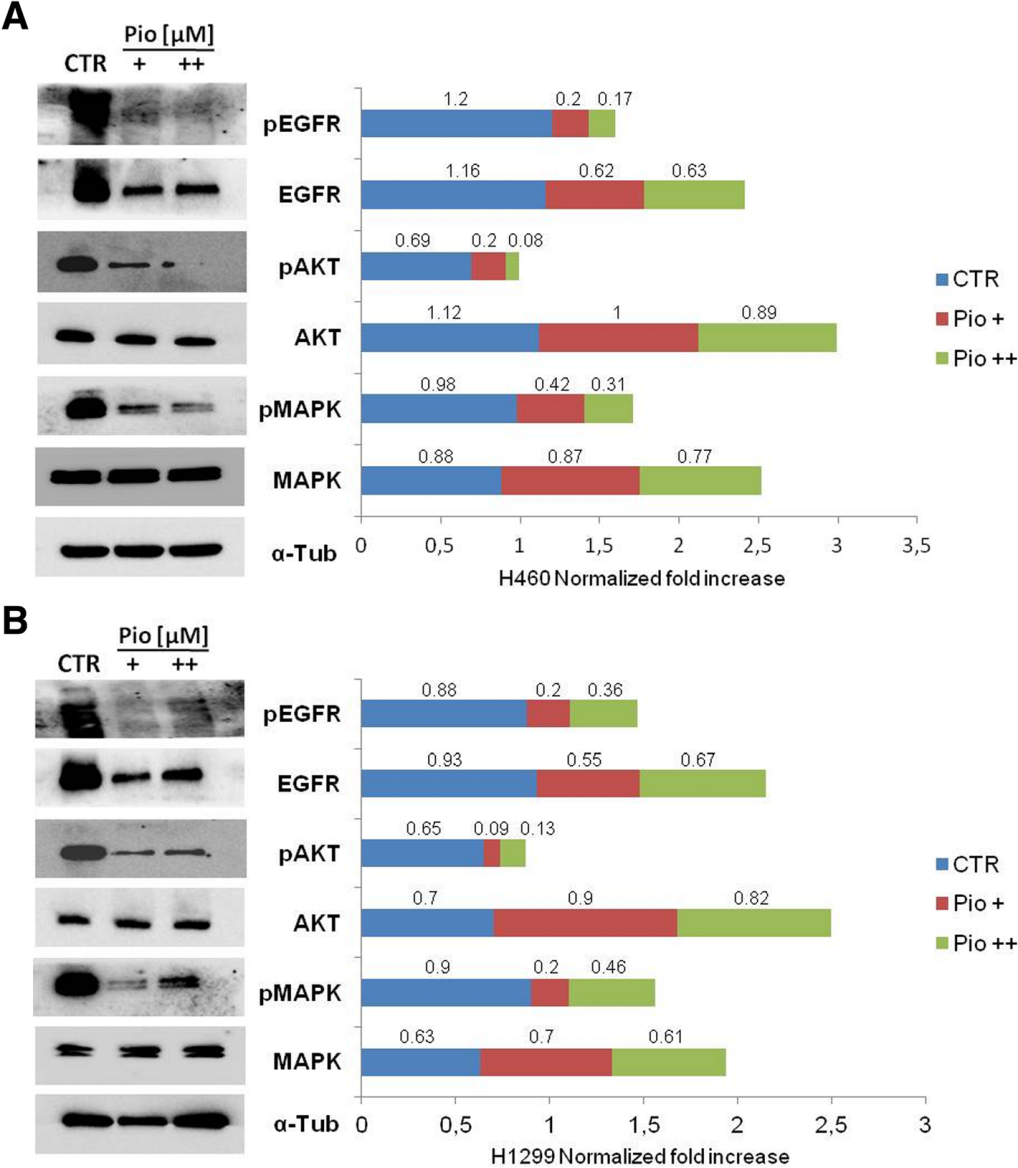
Effect of pioglitazone in anti-proliferative in H1299 and H460 lung cancer cells. Western blot analysis of intracellular proteins EGFR, AKT and MAPK, and their phosphorylated isoforms, were performed on lysates from cells following indicated pioglitazone treatments: +: 1 μM; ++: 10 μM. α-tubulin was included as the loading control. **a** H460 and (**b**) H1299 cell lines

Considering the effect of pioglitazone on in vitro proliferation and the down regulation of specific genes analyzed by microarray (Fig. 4), we selected two significant doses of the drug, 1 and 10 μM, to better investigate the effect of pioglitazone treatment on key intracellular downstream signaling pathways. Western blots were performed to assess total and phosphorylated EGFR and downstream effectors involved in cell survival and proliferating signals (total MAPK, phospho-MAPK, total AKT, phospho-AKT). As shown in Fig. 6, pioglitazone treatment, as single agent, caused a pronounced decrease of activated phosphorylated EGFR, AKT and MAPK levels in both cell lines. These results demonstrate the critical role of pioglitazone as inhibitor of cellular activities linked to growth and survival as well as in pathological contests, such as tumorigenesis and metastasis.

### Effect of pioglitazone on cellular metabolism

**Fig. 7:**
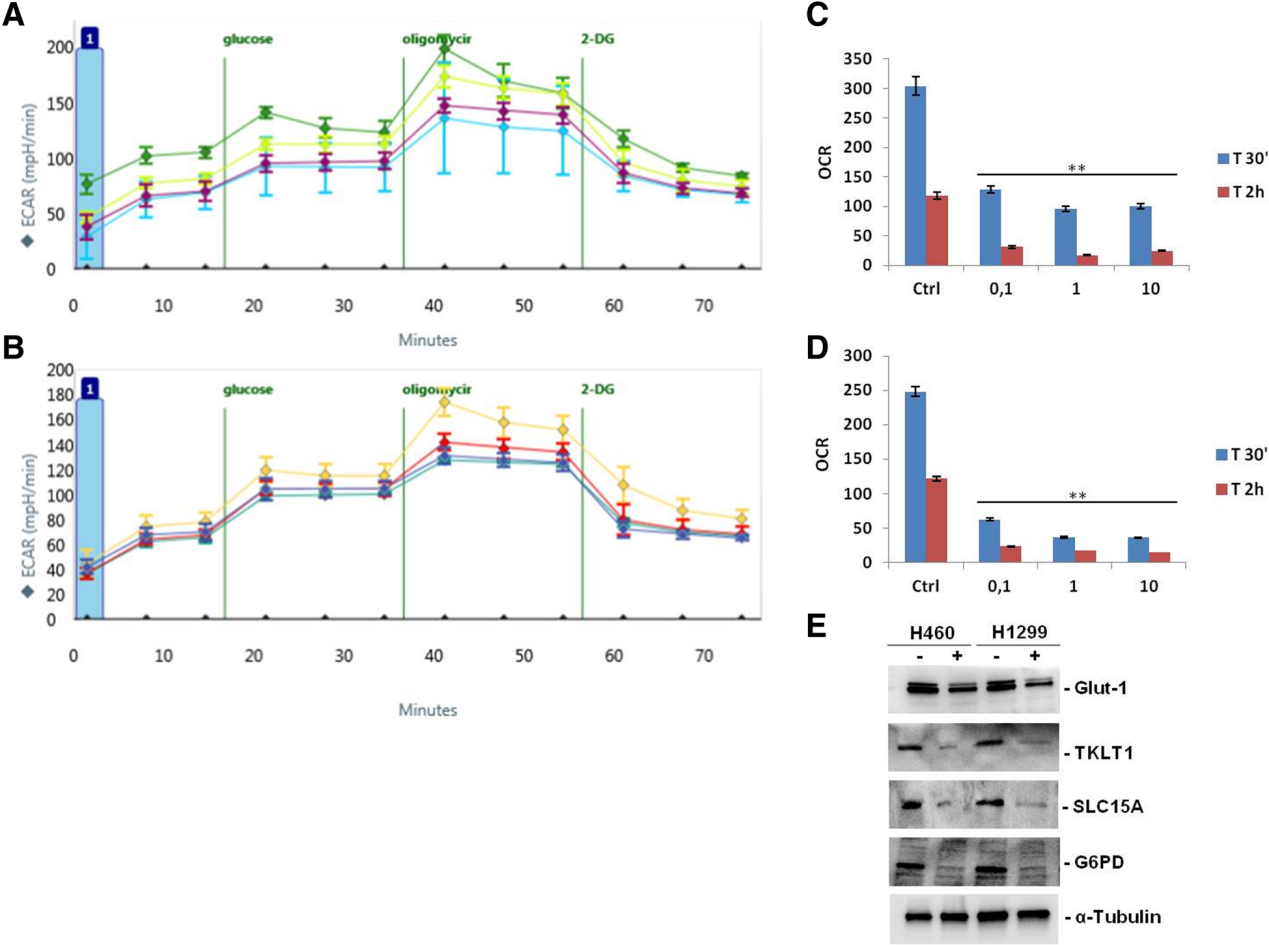
Effect of pioglitazone on cellular metabolism. Analysis of glycolysis by SeaHorse in lung cancer cells following pioglitazone treatment (range 0.1–10 μM) for 30 min and 2 h. **a**, **b** Graphical representation of extracellular acidification rate (ECAR) in H1299 and H460 cell lines: each colored curve indicated concentration of pioglitazone treatment: for H1299 (**a**): green, untreated control; yellow, 0.1 μM; light blue, 1 μM, and purple, 10 μM. For H460 (**b**): yellow, untreated control; red,0.1 μM; light blue,1 μM, dark blue, 10 μM. **c**, **d** Histograms represented the ECAR quantification time dependent, in H1299 (**c**) and H460 (**d**) cell lines. *P* values < 0.05 were considered as statistically significant (**). **e** Expression of Glut-1, TKLT1, SLC15A and G6PD evaluated by immunoblotting in H1299 and H460 cell lines. +: pioglitazone 10 μM. α-tubulin was used as the loading control

Glycolysis is the process by which glucose is broken down into components used for biosynthesis or energy production; activation of this pathway in oxygen-abundant conditions is associated with a variety of cellular functions such as proliferation, immune cell activation, and cancer. In most cancer cell lines mitochondrial activity that leads to CO_2_ production is a potentially significant source of extracellular acidification. In order to examine the effect of pioglitazone treatment on cellular metabolism in human NSCLC, we performed an in vitro experiment treating H1299 and H460 cell lines with different dose of pioglitazone and measuring by Seahorse analysis, the proton efflux (ECAR) as well as mitochondrial O_2_ consumption rate (OCR) to deliver a live-cell bioenergetic profile and detect changes in metabolic function in real time (Fig. 7a, b). We analyzed the respiratory rates at two time points (30 min and 2 h) after treatment and compared with basal values. In both cell lines was observed the same metabolic profile: a decrease in extracellular acidification (ECAR) at both time points in a dose range of pioglitazone from 0.1 to 10 μM with a significant reduction in glycolytic process after 2 h treatment compared to the untreated control (Fig. 7c, d). These results show that pioglitazone exerts its anti-tumoral effects also by acting at metabolic level on the inhibition of glycolysis in cancer cells.

Moreover, to confirm the metabolic effects of pioglitazone we investigated key proteins indicative of altered glucose metabolism in cancer cells, such as the transporters Glut-1 and SLC15A (Solute-linked carrier family A1 member 5), G6PD (glucose-6-phosphate dehydrogenase) and TKTL1 (Transketolase) (Fig. 7e). Glut-1 is a facilitative membrane glucose transporter, which is most frequently implicated in human cancer as main regulatory of glycolysis and SLC15A. SLC15A is a glutamine transporter which plays an important role in metabolism and amino acid homeostasis in a variety of cells and tissues. Following pioglitazone treatment, we detected a decrease of Glut-1 and SLC15A expression thus further confirming the ability to reduce the glycolytic flow and the uptake of the amino acid as the cells tries to maintain homeostasis, in H1299 and H460 cancer cells, respectively. As the over expression of Glut-1 has been reported in various types of malignancies [16], the down regulation of Glut-1 and SLC15A by pioglitazone confirms its antitumor potency on cell growth and further sensitize tumor cells to apoptosis.

G6PD protein works at various stages of cell proliferation through glucose binding and activity, and is the first rate-limited enzyme of the pentose-phosphate pathway. G6PD has been implicated in the regulation of cellular antioxidative mechanisms and the most important physiological function of this protein is to produce NADPH and ribose 5-phosphate. The knockdown of G6PD expression with siRNA decreased tumor cell proliferation and enhanced apoptosis [17, 18].

TKTL1 enzyme reactions is involved in oxygen-independent glucose degradation and plays a crucial role in nucleic acid ribose synthesis utilizing glucose carbons in tumor cells. TKTL1 over expression has been associated with the malignant cells and is presumably involved in the metabolic switch leading to this glycolytic tumor phenotype [19]. In our study we observed a significant reduction of both G6PD and TKTL1 after pioglitazone treatment according to their role in cancer cells that often lose the balance of oxidation and antioxidant.

## Discussion

Several preclinical studies have shown that antidiabetic drugs can change the risk of multiple cancers. Insulin sensitizers TZD, known as PPAR-γ agonists, represent one of the antidiabetic drug options to directly reduce insulin resistance in patients with diabetes mellitus. PPAR-γ ligands such as TDZs can affect mitogen-activated protein Kinase (MAPK) cascade, which plays a central role in intracellular signaling. Other evidences showed that troglitazone induces apoptosis of lung cancer cell line NCI-H23 via a mitochondrial pathway through the activation of ERK1/2 [12]. In addition to anti-proliferative effects, TZD can sensitize cancer cells to anticancer therapies enhancing the cytotoxic effect of cisplatin and oxaliplatin by suppressing survivin and increasing the apoptosis-inducing factor (AIF) expression [20]. Among TZDs, pioglitazone was approved for the treatment of type 2 diabetes and continues to be recommended in current guidelines since pioglitazone bladder cancer concerns have been largely attenuated by recent evidences [21]. The correlation between pioglitazone and cancer is probably related to regulation of trans-activating genes that regulate cell proliferation, differentiation and apoptosis. Pioglitazone selectively targets the PPARγ target genes and it has anti-proliferative effects in a series of malignancies.

In this study, we investigated the effect of pioglitazone in the human NSCLC and described the mechanisms by which it exerts its antitumor effect. In the first instance, we performed in vitro dose-response treatments with pioglitazone both on lung cancer cells and on normal bronchial cellsand we found that it has anti-tumoral effect in terms of cell proliferation, invasion and migration by observing a dose-dependent inhibition in all cancer cell lines used demonstrating a significant anti-proliferative effect and a strong reduction in cell invasiveness. This trend exclusively occurred in cancer cells rather than in normal cells that appear to be insensitive to pioglitazone treatment. The antiproliferative effect of the pioglitazone was also supported by analysis on patients through the preparation of organoids derived from 3D cultures. In order to deepen the molecular mechanisms underlying this effect, we initially conducted a screening on all genes to analyze changes in gene expression profiles after treatment with pioglitazone. Through this analysis we hypothesized that pioglitazone works by switching off the genes involved in cell proliferation, such as the cascade of MAPK, and, on the contrary, by activating genes involved in the apoptosis process such as the Caspases family. The pro-apoptotic effect of pioglitazone was also confirmed by labeling with annexin V and subsequent protein expression analysis observing an increase in caspase-3 and a reduction in survivin, pro-apoptosis and pro-survival markers, respectively.

Some of TGFβ oncogenic activities are linked to its induction of a phenotypic switch known as the EMT, in which cell adhesions are disrupted, the surrounding matrix is degraded, and the tumor cells become more motile and invasive, thereby increasing their metastatic potential [22]. The over expression of TGFβ ligands has been reported in most tumor types, and elevated levels of these ligands in tumor tissues or in patient serum correlates with more metastatic phenotypes or poorer patient outcome [23]. We asked if pioglitazone influences the TGFβ pathway by regulating the expression of some molecules involved in such signaling. Consistently with this hypothesis, we have shown that the treatment with pioglitazone reduces the expression of the TGFβR1 and consequently it also reduces the expression of the SMADs family members, in particular SMAD3. These data correlate with recent studies suggesting that the majority of TGFβ target genes are controlled through SMAD3-dependent transcriptional regulation [24]. Cancer cells undergo EMT in response to TGFβ1, therefore, inhibition of the TGFβ/SMADs pathway suggests that the effect of pioglitazone on tumor cells interferes with EMT process and distant metastasis spread. To investigate other molecular mechanisms of pioglitazone-induced cell death, we first demonstrated the involvement of apoptosis quantifying annexin V labeled cells and then we analyzed the expression pattern of intracellular markers: in fact treatment with pioglitazone as single agent resulted in a concomitant decrease in the level of protein activated phosphorylated MAPK and AKT suggesting its role in inhibition of cell proliferation.

Furthermore, from the bioenergetic point of view, AKT has been shown to promote the metabolic shift towards aerobic glycolysis through a variety of mechanisms, including the phosphorylation/activation of the main glycolytic enzymes and the induction of glucose transporter expression and their localization to the cell membrane [25]. Therefore, the reduction of phosphorylated AKT levels that we have been observed after treatment with pioglitazone could also result in a reduction in cell metabolism [26, 27]. In fact, the altered glucose metabolism is considered an important hallmark of cancer.

The transition to an energy metabolism largely based on glycolysis, even in the presence of oxygen, leads to a metabolic state also known as the “Warburg effect”: tumors acquire the unusual property of taking and fermenting glucose in lactate in the presence of oxygen (aerobic glycolysis) and consequently the mitochondrial respiration defects represent the link between aerobic glycolysis and cancer [28, 29].

In light of this, we investigated the glycolytic activity of lung cancer cells before and after treatment with pioglitazone. Our results showed that H1299 and H460 cell lines, following treatment, underwent a drastic reduction of the glycolytic process at all doses tested. In particular, the effect of pioglitazone is already significant after 30 min of treatment in both cell lines with an almost 100% reduction of extracellular acidification compared to untreated control cells which maintained their high basal metabolic rate typical of tumor cells. Since tumor tissue is often characterized by decrease of oxygen pressure levels, the ability to utilize glucose in the absence of oxygen represents a selective growth advantage for tumor cells. This mechanism could presumably occur through the involvement of specific molecules as Glut-1, SLC15A. G6PD and TKTL1.

The enhancement of these protein has been reported in various cancers and mediates biologically significant effects. The majority of tumors haveTKTL1 protein up-regulated and GLUT1 expressed simultaneously; indeed, several studies reported that SLC1A5 is expressed in 66% of patients suffering from NSCLC [30] and targeting SLC1A5 in non-small cell lung cancer cells induces apoptotic cell death by impairing their ability to uptake sufficient glutamine from the extracellular environment [31].

Therefore, we analyzed the expression profile of these protein in lung cancer cells treated with pioglitazone.

observing a down regulation of them after pioglitazone treatment. These data support our hypothesis of a possible role of pioglitazone in cancer pathogenesis.

All together the data from the present study demonstrate that pioglitazone, in addition to its effectiveness as an antidiabetic drug, acts as an anticancer agent in NSCLC cell lines through several possible mechanisms. Its action has an anti-proliferative effect on the inhibition of crucial markers of the signal transduction as well as the MAPK/AKT cascade as well as on the TGFβ/SMADs system thus carrying out an anti-metastatic action on the inhibition of EMT. Finally, the antitumor potency associated with down regulation of Glut-1 could strengthen the contention between cancer and glucose metabolism and offer a new therapeutic option for the treatment of NSCLC. These considerations lead us to consider pioglitazone as anticancer agent and open new possibilities of treatment combination with other drugs.

## Conclusions

In this work, we explored the role of pioglitazone in human NSCLC and described the mechanisms by which it exerts its antitumor effect. The data shown that pioglitazone represent an attractive treatment tool for NSCLC able to act in different molecular pathways: both through the inhibition of growth and invasion of cancer cells (in in vitro and ex vivo models) and through the modulation of bioenergetics and cancer metabolism. Moreover, our preliminary data about the generation of 3D cultures derived from patient samples, allows us to hypothesize a translational approach that we will elaborate in future works.

## Abbreviations

7AAD: 7-Amino-Actinomicin D
AIF: apoptosis-inducing factor
ECAR: extracellular acidification rate
EMT: epithelial-to-mesenchymal transition
G6PD: glucose-6-phosphate dehydrogenase
IC_50_: concentrations inhibiting 50%
MTT: 3-(4,5- dimethylthiazol-2-yl)-2,5 diphenyltetrazolium bromide
NSCLC: non small cell lung cancer
PPAR: peroxisome proliferator activated receptor
SD: standard deviation
SLC15A: Solute carrier family 15
TKLT1: transketolase
TZDs: thiazolidinediones
